# Clinical and histopathological study of the oral multifocal melanoacanthoma: A case report

**DOI:** 10.4317/jced.55344

**Published:** 2019-04-01

**Authors:** Ingrid-Morgana-Fernandes Gonçalves, Daliana-Queiroga-Castro Gomes, Jozinete-Vieira Pereira, Cassiano-Francisco-Weege Nonaka, Pollianna-Muniz Alves

**Affiliations:** 1 DMD, MSc. Postgraduate student, Department of Dentistry, State University of Paraíba, Campina Grande, Paraíba, Brazil; 2 MSc, PhD. Professor Department of Dentistry, State University of Paraíba, Campina Grande, Paraíba, Brazil

## Abstract

Melanoacanthoma is a blackened mucocutaneous lesion, mainly affecting individuals with dark skin and exhibiting rapid development. Differential diagnosis includes nevus, amalgam tattoo and melanoma. This article reports a case of a 53-year-old white woman, who exhibited multiple blackened lesions on the gingiva and upper lip. After incisional biopsy, the presence of numerous melanin-containing dendritic cells distributed throughout the epithelial thickness, which were S-100 (+), were observed microscopically. Final diagnosis was multifocal oral melanoacanthoma. Follow-up for 28 months has shown appearing of more lesions in gingiva and upper lip. Therefore, the importance of differential diagnosis of oral melanoma with the group of oral pigmented lesions, and possible associated systemic diseases were evaluated.

** Key words:**Melanocytes, pigmentation, oral mucosa, gingiva, differential diagnosis.

## Introduction

Cutaneous melanoacanthoma was described for the first time by Bloch, in 1927, however, the term melanoacanthoma was introduced by Mishima and Pinkus in 1960 (1), observing that this lesion could occur in both the skin and mucosa, such as those of the oral cavity. The first case of oral melanoacanthoma (OMA) was published in 1981(2).

OMA mainly occurs in dark-skinned women, in the age range between the third and fourth decade of life (1-3). Pathogenesis of this lesion is uncertain, although its clinical behavior suggests a reactive process (3), with its possible regression being observed after removal of the causal factor (1). Characterized as being a rare, well-circumscribed, pigmented lesion representing approximately 0.09% of melanocytic lesions, of brownish to blackish color that may present as a single lesion, and less frequently as multiple lesions (2,4). The most frequent intraoral sites are the jugal mucosa (51.4%), palate (22.2%), lip (15.2%), and gingiva (5.6%) (5). This circumstance may explain the high incidence of OMA in mobile mucosa that is more vulnerable to trauma (2).

Clinical differential diagnosis includes nevus, amalgam tattoos and melanoma. In view of the foregoing, this paper aimed to relate a clinical case of multifocal OMA, emphasizing its clinical and histological aspects, and possible differential diagnoses.

## Case Report

**Figure 1:**
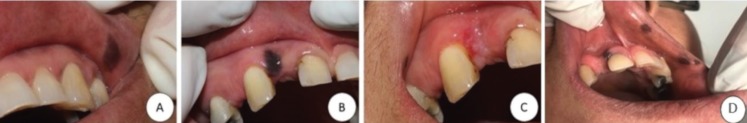
Clinical aspect of OMA. A) Brownish macula with precise limits localized in the upper lip region on the right side. B) Presence of blackish macula in the maxillary anterior region. C) Brownish macula of smaller size, localized in the upper lip region on the left side. D) Final clinical aspect of OMA after 28 months of follow-up of patient. Three brownish macula localized in the upper lip on the left side, and presence of other blackish macula in the gingiva anterior region.

**Figure 2:**
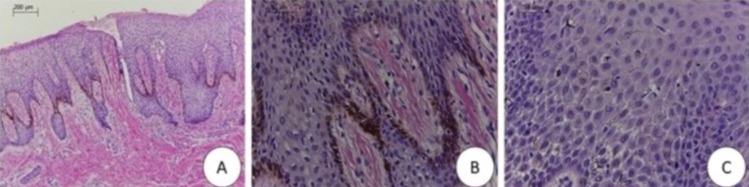
A) Photomicrograph, at lower magnification, exhibiting epithelium with evident acanthosis, and melanocytic cells with brownish cytoplasmic pigments localized throughout the entire basal extension (HE, 100X). B) Photomicrograph, at higher magnification, showing evidence of brownish cytoplasmic pigments, compatible with melanin, restricted to the melanocytic cells (HE, 400X). C) Photomicrograph, exhibiting spongy focal areas and the presence of numerous dendritic cells that contained brownish pigments in their cytoplasm, dispersed throughout the entire extension of the epithelial tissue (HE, 400X).

**Figure 3:**
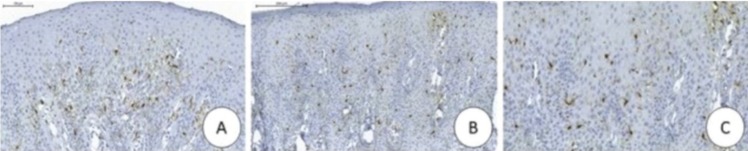
A) Photomicrograph exhibiting immunoexpression of S-100 in the dendritic cells dispersed throughout the entire extension of the epithelial tissue (ADVANCE, 40X). B and C) Photomicrographs, at higher magnification, showing evidence of immunopositivity of S-100 and confirming melanocytic presence (ADVANCE, 100X and 400X).

The patient, a 53-year-old white woman, sought the stomatology service with chief complaint of “dark stains in the mouth” (SIP) that had appeared approximately one year previously. On physical intraoral exam, presence of brownish and blackish macula with a smooth, non-ulcerated surface was observed, localized in the maxillary right gingival mucosa, and on the internal mucosa of the upper lip (Fig. 1). Patient presented no black stains on the skin, denied use of medications, and had no associated systemic disease. She reported being an ex-smoker since a year ago. Based on clinical findings, diagnostic hypotheses were melanocytic macula or nevus, and all the pre-operative exams requested were found to be normal. After incisional biopsy of the lesion in the maxillary anterior gingival region, microscopic findings revealed a fragment covered with parakeratinized stratified pavimentous epithelium, exhibiting acanthosis and elongated epithelial projections. In the basal layer and more superior layers, the presence of dentritic cells containing brownish cytoplasmic granules compatible with melanin were observed. The adjacent fibrous connective tissue exhibited subepithelial melanophages and slight mononuclear inflammatory infiltrate. These findings were consistent with OMA (Fig. 2). Immunohistochemistry with S-100 revealed immunopositivity for dendritic cells disposed throughout the entire extension of the epithelium, thus confirming conclusive diagnosis of OMA (Fig. 3). After two years 28 months of follow-up, the patient has presented development of other brownish and blackish macula in gingiva and upper lip.

## Discussion

OMA is a rapid growing lesion occurring mainly in black individuals, and in the majority of cases, in women (2.1:1), with a mean age of 28 years (2,5,6). Thus, differing from the case here related, in which the patient was white. Brooks *et al.* (7) emphasized that only 10% of cases of OMA were observed in white individuals. In decreasing order, this lesion affects the jugal, labial, palatal and alveolar mucosas. In the present case, gingiva and upper lip were affected, bilaterally. According to Tapia *et al.* (6), gingiva is outstanding as an atypical site for OMA. According to a previous study by Brooks *et al.* (7), only 18.9% of the patients with OMA present multiple lesions. Thus, they show evidence of peculiar clinical characteristics of the case here related. In order to review of the literature, it was made a table with a summary of prior reported cases of OMA ( Table 1), published until 10th January 2019. With respect to cutaneous melanoacanthoma, in the majority of cases, this presents as multiple lesions, preferentially localized in the regions of the head, neck and thorax, and with lower frequency in the eyelids or external region of the lips (1).

**Table 1:**
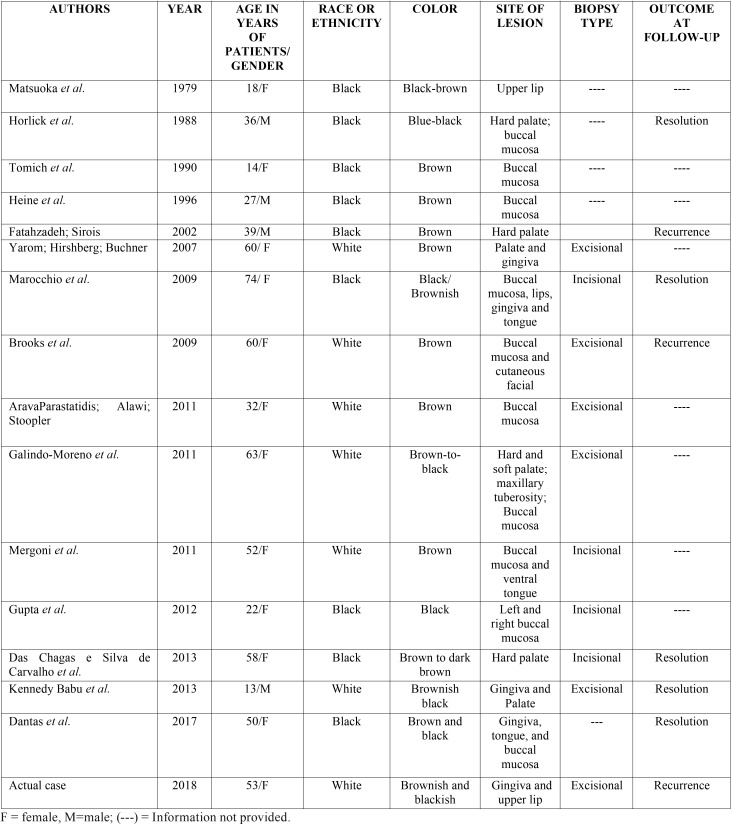
Summary of all cases of oral melanoacanthoma published on literature with their respective characteristics.

Etiopathogenesis of OMA is still uncertain, relating it with a reactive process. Gondak *et al.* (3) mentioned that the proliferative activity of the melanocytes was associated with a continuous traumatic stimulus, and that after eliminating the irritants, or biopsy, they tended to regress spontaneously, differing from the cutaneous melanoacanthoma in which the lesion persists, even after intervention. This factor strengthens the reactive etiology of OMA (1,5). In the case here related, the patient did not inform any possibly associated traumatic factor. Therefore, we suggested a possible idiopathic origin of OMA in our patient.

Clinical differential diagnosis of pigmented oral lesions includes physiological melanic pigmentation, smoker’s melanosis, melanoma and nevus. Physiological pigmentation occurs due to increase in the production of melanin and not due to increase in the number of melanocytes, as occurs in OMA (2). Whereas, in melanosis associated with smoking, the intensity of pigmentation is time and dose-dependent, differently from OMA, in view of the rapid development of the lesion. Melanoma is characterized by the atypical proliferation of melanocytes, a fact that does not occur in OMA (3,4). Melanocytic nevus exhibits nevus cell proliferation, a characteristic not found in OMA (2,4). This increase in melanin deposition could also be attributed to Addison’s disease, hyperparathyroidism and the McCune-Albright and Peutz-Jeghers syndromes (8,9). However, our patient presented no associated change of a systemic order.

For the conclusive diagnosis of OMA, biopsy is fundamental, and the morphological findings are most characteristic, exhibiting numerous, strongly pigmented melanocytes throughout the epithelium, with evident acanthosis. Melanin is generally restricted to the melanocytes, while the adjacent keratinocytes do not contain pigment (1-3,6). These were characteristics shown in the case presented here. Immunohistochemical analysis was performed, but it was not imperative for diagnosis (3). Some immunohistochemical studies habitually show evidence of diffuse nuclear and cytoplasmic immunoreactivity of the dendritic melanocytes to protein S-100, HMB-45 and melan-A in OMA (2,3,7). In this case, protein S-100 was used, and its immunostaining confirmed the presence of melanocytes, thus being in agreement with previous researches.

Therefore, based on the findings discussed in the literature, OMA is a rare condition that must be distinguished from other diffuse pigmentations. Its association with other systemic changes must always be investigated, since oral pigmentations are presented in various clinical patterns that may vary right from physical changes only through to oral manifestations of systemic diseases and malignancies.
